# Human Polyomaviruses (HPyV) in Wastewater and Environmental Samples from the Lisbon Metropolitan Area: Detection and Genetic Characterization of Viral Structural Protein-Coding Sequences

**DOI:** 10.3390/pathogens10101309

**Published:** 2021-10-12

**Authors:** Ana Carolina Condez, Mónica Nunes, Andreia Filipa-Silva, Inês Leonardo, Ricardo Parreira

**Affiliations:** 1 Unidade de Microbiologia Médica, Instituto de Higiene e Medicina Tropical (IHTM), Universidade Nova de Lisboa (NOVA) Rua da Junqueira Nº100, 1349-008 Lisboa, Portugal; a.condez@campus.fct.unl.pt; 2 iBET, Instituto de Biologia Experimental e Tecnológica, Apartado 12, 2781-901 Oeiras, Portugal; mnunes@ibet.pt (M.N.); andreiasilva@ibet.pt (A.F.-S.); ines.leonardo@ibet.pt (I.L.); 3 ITQB NOVA, Instituto de Tecnologia Química e Biológica António Xavier, Universidade Nova de Lisboa, Av. da República, 2780-157 Oeiras, Portugal; 4 Global Health and Tropical Medicine (GHTM) Research Centre, 1349-008 Lisboa, Portugal

**Keywords:** human polyomavirus, wastewater, environmental water, multiplex-PCR, phylogenetic analysis, Portugal

## Abstract

Due to the lack of reliable epidemiological information regarding the geographic distribution and genetic diversity of human polyomaviruses (HPyV) in Portugal, we addressed these issues in this initial study by focusing on the Lisbon Metropolitan area, the most populated and culturally diverse hub in the country. The HPyV structural protein-coding sequence was partially amplified using two touch-down PCR multiplex protocols, starting from water samples, collected between 2018 and 2020, where viral genomes were detected. The obtained results disclosed the frequent detection of HPyV1, HPyV2, HPyV5, and HPyV6 in 35.3% (*n* = 6), 29.4% (*n* = 5), 47.1% (*n* = 8) and 29.4% (*n* = 5), respectively, of the water samples analyzed. The sequences assigned to a given viral species did not segregate to a single genotype, this being especially true for HPyV2 for which five genotypes (including a putative new genotype 9) could be identified. The phylogenetic trees obtained for HPyV5 and HPyV6 had less resolving power than those obtained for HPyV1/HPyV2, but both viruses were shown to be genetically diverse. This analysis emphasizes the epidemiological helpfulness of these detection/genetic characterization studies in addition to being relevant tools for assessment of human waste contamination.

## 1. Introduction

Polyomaviruses are small (~50 nm) non-enveloped viruses with circular double-stranded genomes with approximately 5 kbp, that infect mammals (humans included) and birds [1]. The International Committee for the Taxonomy of Viruses places them within the *Polyomaviridae* family (Order *Sepolyvirales*), where they are distributed into six genera, encompassing a total of 112 viral species [2]. At least 12 of these species are considered bona fide human polyomaviruses (HPyV), and these have been placed in three genera (*Alpha*-, *Beta*-, and *Deltapolyomavirus*), defined as a function of phylogenetic relationships established based on the analysis of the large T antigen [2,3].

The high global seroprevalence regarding the great majority of HPyVs suggests they infect humans from an early age [4,5]. However, despite being true human pathogens, the diseases caused by HPyVs are usually associated with diverse stages of immunosuppression, either in senior individuals, those infected with the human immunodeficiency virus (HIV), or undergoing chemotherapy, or in association with organ transplants [6,7]. Four of these viruses have been linked with diseases that may be serious/life-threatening, and which include hemorrhagic cystitis in patients who have undergone bone marrow transplantation (caused by HPyV1/BK polyomavirus), progressive multifocal leucoencephalopathy (PML) associated with AIDS (caused by HPyV2/JC polyomavirus), Merkel-cell carcinoma, an aggressive cell tumor caused by HPyV5 (or MC virus), or trichodysplasia spinulosa, a rare skin disease caused by HPyV8 (or TS polyomavirus) [8] (Supplementary Table S1).

Human polyomaviruses are frequently found in excreta as it has been repeatedly demonstrated since the isolation of the first (named HPyV1 or the BK polyomavirus) from urine in the early 1970s [9]. Indeed, it has become increasingly apparent that they are present in the environment all year-round, which supports their use as indicators of human waste contamination. Human health risk assessment regarding the presence of fecal contaminants in environmental waters has traditionally been based on bacterial counts and especially on *Bacteroides* sp., which outnumber conventional fecal indicator bacteria (FIB), such as coliforms and enterococci [10,11,12]. However, several human viruses, including adenoviruses, aichiviruses, and human polyomaviruses have been viewed as better options for human enteric contamination assessment due to their host-specificity, non-seasonal distribution, and high titers in both influent (i.e., untreated sewage) and environmental water samples [13].

The lack of consistent epidemiological data regarding the geographic distribution, and genetic diversity, of human polyomaviruses in Portugal, has prompted us to approach this study from a molecular epidemiology perspective. Indeed, molecular epidemiology studies based on the analysis of viruses (most frequently their genomes) frequently disclose genetic diversity and population fluctuations and allow an assessment of the viral factors that may be associated with different outcomes in virus–host interactions [14].

Due to logistic constraints associated with the COVID-19 pandemic, this exploratory study was carried out from the analysis of water samples that were obtained in the Lisbon metropolitan area (LMA), where northern and southern areas are separated by the Tagus River. For this purpose, two touch-down nested-PCR multiplex protocols were developed for the detection of HPyVs, not only from raw sewage but also from environmental waters (estuary/river/rivulet) samples. This was followed by their genetic analyses mostly focused on DNA sequencing of individual plasmid recombinant molecules, but further complemented with Next Generation Sequencing (NGS) data. When combined with phylogenetic analyses, our data demonstrated the circulation of diverse strains of HPyV1, HPyV2, HPyV5, and HPyV6.

## 2. Results

### 2.1. Primer Design and Touch-Down PCR Setup

**Figure 1:**
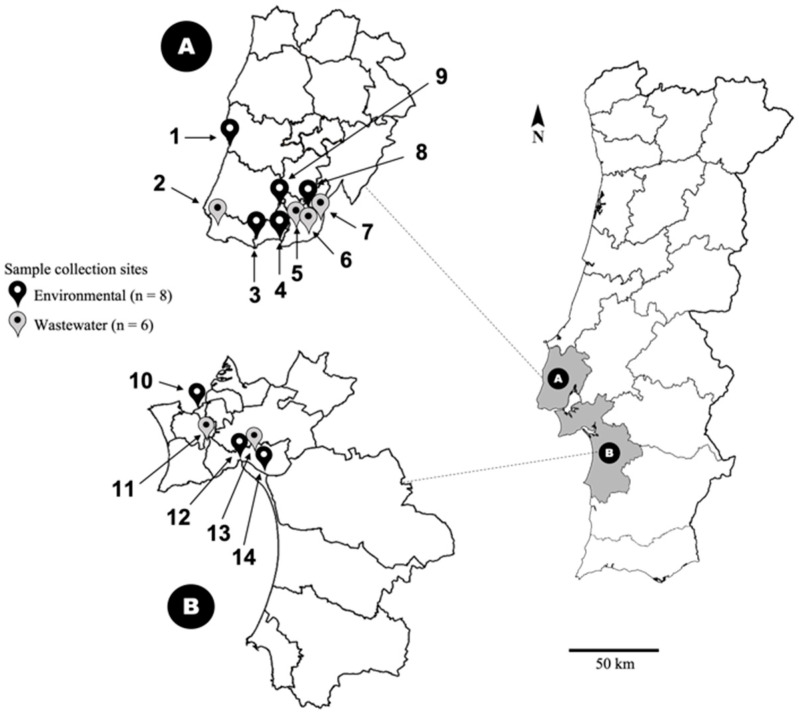
Geographic distribution of the wastewater and environmental sampling sites (numbered from 1 to 14). The areas indicated by (**A**) (sites 1 to 9) and (**B**) (sites 10 to 14) correspond to the two largest administrative districts of the LMA that are separated by the Tagus River and its estuary. The number of wastewater and environmental sampling sites is indicated between brackets.

The work carried out in this report was based on the detection and genetic analyses of partial HPyV genomic sequences amplified from both wastewater (raw sewage; *n* = 9) and environmental (estuary/river/rivulet; *n* = 8) samples (n_total_ = 17) collected at 14 sites in LMA (Figure 1), within a three-year window frame, from 2018 to 2020. Because of the exploratory nature of this analysis, the water samples used originated from a relatively small number of sites. Viral-like particles were concentrated from these samples using the skimmed milk flocculation protocol, and the HPyV structural protein-coding region was targeted for amplification. A preliminary analysis of the complete structural-protein coding regions using reference sequences downloaded from GenBank (Supplementary Figure S1) indicated that viral sequences were distributed into two large monophyletic clusters. One of these included HPyV3, HPyV4, HPyV6, HPyV7, and the so-called Quebec polyomavirus (QPyV) [15], while a larger cluster grouped HPyV1, HPyV2, HPyV5, HPyV8, HPyV9, HPyV10, HPyV11, HPyV12, HPyV13, and HPyV14. The observed distribution of HPyV sequences into two separate phylogenetic lineages (Lineage-A/L-A and Lineage-B/L-B) guided the design of amplification primers, using lineage-specific multiple sequence alignments (MSA). However, sequences from HPyV12, HPyV13, HPyV14, and QPyV were not considered for primer design due to their low prevalence and/or uncertainty around their assignment as human-specific viruses [16,17,18].

The PCR amplification primers used during this study were defined using software tools and visual inspection of MSA of the structural-protein coding regions of the HPyV segregating into L-A and L-B (Table 1), and aimed to obtain segments with approximately similar, though not identical, sizes. These primers were then used to define two multiplex nested-PCR approaches for HPyV amplification, which were designated PCR-A and PCR-B, respectively for amplification of genomes clustering within L-A and L-B. As indicated in Table 1, each round of PCR-A included a total of 8 types of primers for the amplification of HPyV1/HPyV2, HPyV5, HPyV8/HPyV9, and HPyV10/HPyV11, while for PCR-B a smaller number of primers was used (*n* = 4) to target the amplification of HPyV3/HPyV4, HPyV6/HPyV7. Furthermore, when aligned to a reference HPyV sequence, the designed primers showed none to minimal (*n* = 1) mismatched to a homologous reference, but higher (from 2 to the total length of the primer) with a heterologous HPyV (Table 1).

Due to the large number of primers used, and the complexity of the template samples (which contained DNA from multiple sources), many possible competitor sequences may hybridize with the primers used. Therefore, a touch-down PCR protocol was chosen to increase both the specificity and sensitivity of the anticipated amplification [19]. In a preliminary experiment, we assessed the performance of a multiplex versus a singleplex approach using primers HPyV1/2Fo and HPyV1/2Ro (1st round) and HPyV1/2Fi and HPyV1/2Fi (2nd round) and a diluted extract of HPyV2 DNA (clinical isolate) as a positive control. Not surprisingly, the obtained results showed that despite using a nested-PCR approach, the expected 1 kb DNA (approximate size) fragment could be detected after the 1st round of amplification in the singleplex format, but only after the 2nd round in the multiplex format. This seems to indicate a loss in the detection sensitivity when multiple primers were combined in a single amplification reaction (Supplementary Figure S2).

The detection of polyomavirus DNA was deemed successful whenever an expected-sized amplicon was observed on agarose gels. For PCR-B, only the HPyV6/HPyV7 primer combination produced amplification specific products after the 2nd round, with no spurious amplicons ever been observed. On the contrary, PCR-A sometimes yielded non-specific amplification products that could not be eliminated with varying amounts of input DNA. Altogether, from a total of nine wastewater and eight environmental samples, HPyV DNA was detected from ten of these (58.8%). However, and as expected, HPyV DNA was consistently detected in sewage samples (100% amplification success) but not from environmental samples (12.5% success), most probably reflecting a considerable difference in viral titer between both sample types.

### 2.2. Analysis of PCR Amplification Results

**Figure 2:**
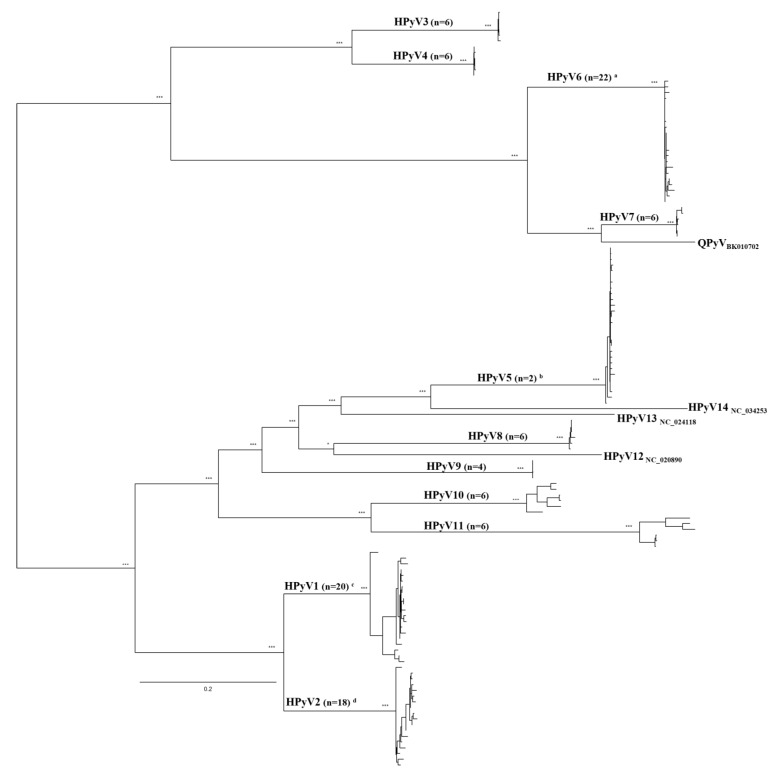
Phylogenetic analysis of HPyV sequences (maximum likelihood) considering a structural protein-coding genome fragment with a minimum of 650 nt. At specific branches, the identity of the HPyV monophyletic clusters is indicated. The number of sequences in each of these is indicated in brackets. The ^a^ (*n* = 16), ^b^ (*n* = 23), ^c^ (*n* = 14), and ^d^ (*n* = 12) in superscript refer to the number of sequences obtained in this study. For HPyV12, 13, 14, and the QPyV the reference sequences are indicated as isolated branches, where the subscripts indicate their respective accession numbers. At the main tree branches, the number of “*” indicates the support revealed by the different phylogenetic reconstruction methods used, assuming as relevant aLRT and bootstrap values ≥75%, as well as posterior probability values ≥0.80 when a Bayesian approach was further used to confirm the proposed phylogenetic clustering. The bar indicates the average number of substitutions per site.

Within the geographic range this study focused on, and considering the heterochronous sampling strategy used, the amplification products resulting from either PCR-A or PCR-B were anticipated not to conform to a given HPyV sequence type. Therefore, the genetic complexity of the obtained amplicons was investigated using an approach essentially based on DNA cloning and analysis of a total number of 73 sequences, most of which (*n* = 68) were obtained from the analysis of independent recombinant plasmids. Four of the total sequences were obtained as a result of NGS analysis of a pool of PCR products (see below), while the remainder corresponded to an amplicon obtained using only HPyV5-specific primers. The obtained sequences were subsequently genetically typed using BLASTn (all of them), and phylogenetic analysis (for those with sizes >650 nt). Altogether, the obtained results disclosed the presence of HPyV1, HPyV2, HPyV5 and HPyV6 in 35.3% (*n* = 6), 29.4% (*n* = 5), 47.1% (*n* = 8) and 29.4% (*n* = 5) respectively, considering the number of water samples analyzed (Figure 2). On the other hand, when the analysis was performed considering the number of obtained sequences, those corresponding to HPyV1, HPyV2, HPyV5, and HPyV6 accounted for 20.55% (*n* = 15), 21.92% (*n* = 16), 35.62% (*n* = 26), and 21.91% (*n* = 16), respectively (Table 2). The process of assigning a viral sequence to a given species (e.g., HPyV1, HPyV2, and so on) was supported by the high phylogenetic signal of the dataset used (which resolved 95.60% of the random sequence quartets using likelihood mapping). This distribution of the amplified sequences into four viral species was further confirmed by NGS sequencing using a pooled library of the 10 PCR-A and PCR-B amplicons obtained from seven samples (six wastewater/one environmental), collected in 2018, 2019, and 2020. From a total of 496,197 HPyV-specific short-sequence reads (accounting for 86.0% of the total number of reads analyzed), 148,851 (30.0%) were assigned to HPyV1, 111,781 (22.5%) to HPyV2, 201,255 (40.6%) to HPyV5, and 34,310 (6.9%) to HPyV6. High coverage and full-length sequencing of the PhiX174 used as a control supported the distribution of the HPyV sequences. Indeed, the qualitative distribution of viral types was the same as the one obtained for individual plasmid analysis. However, many factors may have contributed to the numerical difference in the distribution of the different HPyV types when the results of the two approaches are compared. Curiously, from the 69 sequences corresponding to either PCR amplicons or recombinant plasmids, and for which the sequence at the amplicon ends could be inspected, nine (13.0%; *n* = 4 HPyV1; *n* = 3 HPyV2; *n* = 2 HPyV5) had been amplified with a priori unexpected primers combination, where one of them was heterologous when considered the type of the sequence in question (e.g., HPyV1 amplified with primer HPyV1/2Fi and HPyV8/9Ri).

For a selection of DNA extracts prepared from two wastewater and two environmental samples from which no HPyV had been found using the multiplex approach, a singleplex amplification assay was conducted to investigate whether the amplification performance would be impacted (Supplementary Table S1). Hence, from the totality of the analyzed samples, only one of them persisted as negative for every reaction while for the remainder, the individual amplification reactions revealed the detection of HPyV1 and/or HPyV2, HPyV5, or HPyV6 DNA. Although no other HPyV was identified, the detection of at least one of the previously detected HPyV in three of the four samples seems to indicate that the use of multiple pairs of possibly competing primers in the multiplex format reduces the sensitivity of the amplification. Indeed, for at least one of the samples, and as the use of control DNA had already shown, the singleplex format may reveal amplification products in the first round of amplification, whereas these are only seen in the second round in the multiplex amplification format. Finally, while the obtained sequences allowed for their genetic characterization using multiple tools, the size of their corresponding amplicons, always above 1 kbp especially in the first amplification round, might have compromised the efficiency of detection of some viral sequence types compared to others.

Although the number of sequences obtained for each virus type was not the same, their analysis revealed considerable genetic heterogeneity for each virus type. Furthermore, the construction of species-specific phylogenetic trees was characterized by the asymmetrically different phylogenetic signals of the HPyV1 and HPyV2 datasets (90.22% and 89.62%) versus the HPyV5 and HPyV6 datasets (47.56% and 43.22%), which indicated that for the latter, the clear-cut definition of genetic lineages may not always be devoid of ambiguities and should be verified with different genetic analyses tools.

### 2.3. Genetic Characterization of Viral Sequences

Phylogenetic analyses carried out using a combination of maximum likelihood and a Bayesian approach (Supplementary Figure S3) showed that HPyV1 segregated into four major lineages, while for HPyV2, seven major clusters, including Portuguese sequences, were identified (Table 2). For both HPyV1 and HPyV2, one of the sequences failed to conform to a previously identified genetic subtype (LC636403 and LC636376, respectively). Therefore, for HPyV1, LC636403 has been characterized as a subtype I-like sequence, while for HPyV2, the singularity of one of LC636376, which was further confirmed by the construction of NNn and by PCOORD (Supplementary Figure S4), lead us to suggest it might correspond to a previously undescribed genotype 9. Overall, for HPyV1, Ib1 was the most frequently identified viral subtype encompassing over 73% of the HPyV1 sequences, while for HPyV2, the most diverse of the virus types identified, an even number of sequences were ascribed to genotype 1 (subtype A and B), 2 (subgroup A2), and 4 (Table 2). For HPyV5 most of the sequences (>65.3%) fell into the so-called Europe/North American cluster, as defined by Martel-Jantin et al. [20]. Due to a comparatively sparser representation of HPyV6 sequences in the public databases, the topological stability of the obtained trees did not support the unambiguous identification of individual genetic lineages (when compared to HPyV1, HPyV2, and HPyV5 trees). However, while the phylogenetic tree suggested a considerable heterogeneity of the 16 sequences obtained, this heterogeneity is somewhat abated in the corresponding NNn, and especially by PCOORD analysis, in which the great majority of the obtained sequences clustered together (with a near-neighboring LC636346), with only one of them (LC636383) segregating away from all the others (Supplementary Figure S4). Finally, the more divergent or single-segregating branches in the phylogenetic trees were investigated by recombinant detection tools, but in no case was there any evidence that they might correspond to intragenic mosaic sequences.

For most monophyletic clusters identified in the obtained phylogenetic trees (Supplementary Figure S3), the sequences associated with them were obtained from samples collected in the same year, either in the same or neighboring sampling points. When multiple sequences were obtained from the same sampling site, they did not necessarily cluster together but rather associated with different references along the branches of their respective phylogenetic trees (Supplementary Figure S3).

## 3. Discussion

In this study, we addressed the detection and the genetic diversity of HPyV circulating in the LMA area using wastewater and environmental water samples. Since Lisbon is the capital city of Portugal and the country’s major economic, social, political, and cultural activity hub, its metropolitan naturally hosts residents from very different cultural and ethnic backgrounds. In addition, some of the samples analyzed in the course of this work had been collected before the onset of the COVID-19 pandemic, when the area was one of Europe’s most valued tourist spots, visited by thousands of tourists annually. When considered together, these conditions were expected to translate into a non-homogeneous distribution of HPyVs.

The experimental approach undertook in this study was based on the design of two-touchdown nested-amplification protocols aiming at the detection of part of the viral structural protein-coding region of a broad-spectrum of HPyVs genomes. While the taxonomic differentiation of HPyVs is, by norm, based on the analysis of alignments of the primary sequence of the large T antigen [3], different studies have also disclosed the genetic resolution capability of the structural protein coding section of the HPyV genome [21,22,23,24,25]. The analysis of this region has been the base for the definition of multiple viral genotypes and subtypes [14], especially for the most systematically identified viruses, that include HPyV1 (or the BK polyomavirus), HPyV2 (or the JC polyomavirus), and HPyV5 (or the Merkel cell carcinoma polyomavirus). These expectations were further confirmed when the analysis of the phylogenetic signal of a dataset containing many representative reference sequences from all viral species (in addition to those obtained in this study) was found to be very high, with 95.60% of the randomly sampled sequence quartets being totally resolved (by likelihood mapping analysis).

Strategies of HPyV detection and analysis as the one here described have been previously used for environmental surveillance of HPyVs, repeatedly demonstrating the usefulness of human polyomaviruses as viral indicators of human waste contamination [13,26,27,28,29,30,31,32,33]. Indeed, their detection allows for the so-called microbial source tracking [34] which corresponds to an effort to track the most frequent sources of fecal contamination (human or animal) in environmental waters for human health risk assessment. While it has relied mostly on the use of *Bacteroides* spp. bacteria as indicators of fecal contamination [10], not only are they not exclusive human bacteria, but they may also multiply in the environment (especially in warmer climates), both of which are undesirable features for a reliable indicator of human-associated fecal pollution [35].

When combined with multiple approaches of DNA sequencing and their analysis, our approach allowed the investigation of the molecular epidemiology of HPyVs in LMA, which, to our knowledge, is here addressed for the first time. Its implementation called for the design of pairs of primers that were combined into two PCR protocols for targeted amplification of two groups of HPyV, supported by a preliminary phylogenetic analysis of the structural protein-coding section of viral reference sequences. On the one hand, these two groups included HPyV1, HPyV2, HPyV5, HPyV8, HPyV9, HPyV10, and HPyV11, while on the other HPyV3, HPyV4, HPyV6, and HPyV7. While a similar strategy had been previously used [33], the strategy here described differs from the latter as it does not allow for preliminary identification of the viral origin for the obtained amplicons as a function of their size. Indeed, the 2nd round amplification products had a priori similar sizes ranging from 881 bp (HPyV3) to 1175 bp (HPyV10), therefore falling into a relatively narrow size range (although considerably larger than the ones studied by Torres et al. [33]). When combined with complementary genetic analyses techniques (including phylogenetic, network, and PCOORD analyses), the experimental approach used in this study supported the detection and unambiguous characterization of different HPyV sequence types. While the sequencing effort was mostly focused on the analysis of independent recombinant DNA molecules with cloned HPyV inserts, it was further supported by an assessment of the genetic diversity of the HPyV types that could be detected by the amplification protocols used, via NGS analysis of a pooled sample of purified PCR fragments.

Whereas different studies have disclosed high rates of HPyV detection, even in environmental samples [26,30,36,37], the detection efficiency of the protocol here used was very high (100%) for wastewater, but considerably lower (12.5%) for environmental samples. Although the ability to detect HPyV genomes with high efficiency from samples collected from environments where these viruses are present in high titers (such as untreated sewage) has been testified in numerous studies carried out over a wide geographic range [30,32,33,38,39,40], curiously, the detection of HPyV genomes in samples collected in western Europe have disclosed lower values (50–75%) even in wastewater samples [26,39]. These different studies most probably reflect differences in virus concentration and PCR amplification, though fluctuations of viral titers in these samples, especially in environmental samples, such as those collected in running rivers/rivulets or estuaries, cannot be discarded. Indeed, the environmental samples analyzed during this study were collected during winter, where the rainwater input is highest, or were under the diluting effect of tides, which contribute to bringing the HPyV titer down.

On the other hand, since HPyV are among the most frequently used human fecal contamination viral indicators [13,31,36,38,41], their presence in raw sewage was expected and could be confirmed in all the wastewater samples used. The most frequently found HPyVs in wastewater are HPyV1 (or BKPyV), HPyV2 (JCPyV), and HPyV5 (MCPyV) [14], and all of them were detected in this study, with HPyV5 accounting for a majority (35.62%) of the obtained DNA sequences. In addition, our analysis also revealed the presence of HPyV6 in wastewater as previously detected [42,43]. Whether the quantitative values calculated for the HPyV types detected truly reflect their distribution in the samples analyzed is open to discussion. Certainly, studies that depend on the efficiency of an in vitro amplification step, may be further biased by subsequent genetic bottlenecks associated with DNA cloning in the experimental workflow, which may further include a non-equivalent and finite analysis of independent clones obtained from each sample. Under these scenarios, the obtained results do not necessarily convey a quantitatively correct idea of the abundance of the different HPyV in each sample. Even the qualitative information obtained may be impacted by the amplification strategy used. Indeed, viruses other than the ones here described (e.g., HPyV3/KIPyV, HPyV10/MWPyV, and HPyV7) have been previously detected in wastewater and/or have been described to be excreted by the urinary or fecal route or due to skin peeling [44,45,46,47,48]. Therefore, their absence from our analysis may reflect (i) their absence, (ii) presence in low titer, or (iii) our inability to detect them. In any case, the independent recombinant plasmid vs. NGS data analysis indicated that the cloning strategy used did not introduce a major skew in our ability to identify HPyV sequences, previously amplified by PCR.

The so-called HPyV1 (also known as BKPyV) has been classified into four different genotypes (I–IV) according to the analysis of the genetic variability of the VP1 coding region [21]. Up to the present day, the majority of the HPyV1 sequences obtained belonged to genotype I, while genotypes II and IV were also detected. Genotype I is distributed worldwide, but its Ib1 and Ib2 subtypes have been associated with South-East Asians and Europeans/West Asians, respectively [49]. The frequent detection of genotype I HPyV1 is followed by that of genotype IV, where the six subtypes (IVa1, IVa2, IVb1, IVb2, IVc1, and IVc2) are prevalent in East Asia, whereas IVc2 is also being found in Europe and Northeast Asia [50]. On a global scale, and in contrast with genotypes I and IV, sequences assigned to genotypes II and III have been more scarcely detected, have a similar distribution, and appear more common in Africans [49]. Although genotype II has been more frequently associated with North/East Africans, their distributions seem to be similar, whereas genotype III has been found mostly in Africans [14].

For HPyV1, phylogenetic and/or sequence similarity searches (using BLASTn) disclosed the distribution of the obtained sequences into genotypes I and III [49]. In genotype I, although most sequences were associated with subtype Ib1, subtype Ia sequences were also detected. Furthermore, a genotype I-like sequence was singled out by phylogenetic analysis (LC636403), but this sequence was associated with genotype I by PCOORD and NNn suggested it most probably corresponds to an Ib1 sequence. Therefore, finding a majority of HPyV1 sequences associated with subtype Ib1 is congruent with the origin of most of the population in LMA. However, one of the sequences clustered among those in genotype III, which is not unexpected due to the extensive number of individuals of African origin living in the regions.

On the other hand, several algorithms have been used for genotyping HPyV2 (also known as JCPyV), allowing its assignment into several different genotypes that, as with HPyV1 genotypes, are usually associated with specific human populations and geographical areas [51,52,53]. Two nomenclatures have been proposed for HPyV2 genotypes, with the one proposed and reviewed by Stoner et al. being the one used in this work [53]. Genotype 1 (further divided into 1A and 1B) is mostly associated with Europeans and North Americans, with genotype 4 registering a similar distribution pattern [52]. On the other hand, genotype 2 has revealed a higher degree of genetic complexity and has, therefore, been classified in five different subtypes: 2A, 2B, 2C, 2D, and 2E. Subtypes 2A and 2C form a paraphyletic group and encompass viral strains linked to Eastern Asians and Native Americans. Subtype 2B is usually associated with Europeans and Eastern Asians, whereas subtype 2D (which is subdivided into 2D1, 2D2, and 2D3) is commonly distributed in Asia [54,55,56]. Lastly, 2E is the subtype that predominates in Oceania [57]. Genotype 3 has been associated with some African and Asian populations, with subtype 3A being most prevalent in South, West, and Central Asia, scarcely in southern Europe [58,59,60], and almost the entirety of the African continent, except for the South, where subtype 3B predominates [54,58]. Likewise, genotype 6 is linked to African populations, is more commonly found in Central and Western Africa [58]. Genotype 7 is classified into subtypes 7A, 7B (encompassing 7B1 and 7B2), and 7C (further divided into 7C1 and 7C2) and is predominantly found in Asia, with subtype 7C also being found in Mauritius [54]. Genotype 8 is the most prevalent in the Western Pacific region, where both 8A and 8B dominate in Polynesia and Malesia, with the last one also being present in Papua New Guinea [57]. Lastly, genotype Eu-c appears to be only present in Northeast Siberia and Japan [52].

In contrast to HPyV1 data, for which most of the viral sequences obtained (73.33%) could be assigned to a single subtype (Ib1), a much more heterogeneous distribution of sequence subtypes was found when we considered HPyV2. Indeed, genotype I (subtypes 1A and 1B Europeans and North Americans), genotype 2 (subtypes 2A2 Eastern Asians and Native Americans and 2B Europeans and Eastern Asians), along with genotype 3 (subtype A South, West, and Central Asia and almost the entirety of the African continent), genotype 4 and a putatively new genotype 9 were also identified. The genetic singularity of the latter was not only suggested by their isolated position in the phylogenetic trees, but also by both NNn and PCOORD analyses. Once again, due to the heterogeneity of the population living in the area where this study was focused, finding a putative new HPyV2 genotype was not unexpected, and formally opens the possibility that other yet undescribed viral variants remain to be characterized.

Based on the analysis of a fragment composed of the large-T antigen coding region and VP1′s coding region, HPyV5 has been divided into five genotypes, each with a strong geographical association: Europe/North America, Africa, Asia, Oceania, and South America [14,20]. Somewhat surprisingly, HPyV5 sequences comprised 35.62% of the total sequences analyzed in this study with most of them (65.38%) clustering among the so-called “Europe/North America” genotype. However, 30.77% of the HPyV5 sequences were also associated with the African genotype. Given the structure of the population living in/visiting Lisbon and its surroundings, the observed distribution was not unexpected. However, one sequence (LC636361) once again failed to cluster inside any of the proposed genetic clusters and radiated away from the African and Europe/North America, and was, therefore, assigned as a member of a broader Europe/North American/African “supercluster”. Its genetic singularity was also confirmed by the NNn analysis (where it could not be associated with any of the groups of references), while it was considered a divergent member of the Europe/North America cluster by PCOORD analysis. Whether this sequence again indicates the existence of a new genetic lineage or its allocation to a specific genetic group in the phylogenetic tree, the result of a low phylogenetic signal of the dataset analyzed remains to be determined in future studies. In any case, additional analyses based on a larger number of samples from different locations most certainly will clarify our understanding of the geographic distribution of this group of polyomaviruses. The latter could also be improved with the analysis of larger, and more polymorphic sequence fragments, or better representative sequence datasets with high phylogenetic-associated signals.

Besides HPyV5, other less frequent HPyVs in waters samples include HPyV6. Due to the still low number of available HPyV6 sequences in databases, studies regarding the molecular epidemiology of this virus remain scarce. Even so, a couple of previous studies have reported the identification of two main clades when analyzing whole-genome sequences and/or partial sequences of HPyV6 [14]. The first group is comprised of HPyV6 sequences from various geographical locations (mostly from North America and Europe, but also including those from Asia and Oceania), while the second group mainly includes sequences of Asian origin. Furthermore, another clustering phenomenon appears to occur inside the first group, where HPyV6 sequences form subgroups according to their geographical origin [14]. However, due to the lack of information available in databases concerning the late region of the HPyV6 genome, we were unable to assess the occurrence of this distribution in this study. Curiously, our analysis revealed an a priori unexpectedly high number of sequences assigned to HPyV6, suggesting that their excretion route is either the feces or urine, with skin-peeling also being a valid hypothesis. Due to the low topological stability of all the phylogenetic trees of HPyV6 sequences, it was not possible to assign them to specific genetic types, although seven of them, obtained from samples collected in 2018 and 2020 at two different collection points, did share a common ancestry, and were assigned to a tree clade also including a sequence from Australia (Supplementary Figure S3). Once again, one sequence (LC636383) diverged from all others in the phylogenetic tree and was associated with different references by NNn and PCOORD. Therefore, as diverse HPyV6 seem to be frequent in LMA, subsequent analyses of these viruses will elucidate their genetic structure.

The use of wide-range viral detection protocols, especially when they explore multiplex amplification strategies, needs to consider the possibility of false-negative detection results, especially when viruses are present in low numbers. Indeed, and as our results have shown, the combination of multiple amplification primers in a single tube may reduce the efficiency of viral detection, especially if single-step (i.e., non-nested) PCR protocols are used. Most protocols reveal the pervasive presence of at least some types of HPyV (including HPyV1, HPyV2, and HPyV5) in wastewater samples where genetic analyses can disclose their genetic diversity, especially in areas where the sewage processing plants received the discharge of sewage effluents from millions of a heterogeneous population. Such is the case of LMA, which has been until recently a major tourist hub and gives residence to native as well as non-native Caucasians (especially from Brazilian and Eastern European ascent), Asian (especially Chinese), and African ancestry (most frequently from the former Portuguese colonies, that include Cape-Verde, Angola, Mozambique, and Guinea-Bissau). Therefore, not only does the detection of HPyV may indicate human waste contamination, the analysis of these viruses (through the study of their genomes) gives important information regarding their molecular epidemiological distribution patterns in the general population. Despite the possible limitations of studies such as the one here described, this study confirms that (i) environmental viral analysis is a powerful tool and that (ii) further methodology developments are still required to fully exploit this research ground.

## 4. Materials and Methods

### 4.1. Virus Concentration by Skimmed-Milk Flocculation and DNA Extraction

All the samples analyzed, corresponding to either solid particle-free wastewater (raw sewage influent) or environmental water samples were collected during October 2018, April 2019, July 2020, and October–November in 2020 in LMA (Figure 1). All samples were brought to the laboratory within <6 h of collection and were immediately stored at 4 °C and processed within 48 h.

Viral-like particles (VLP) were concentrated from 1 L of influent or 10 L of environmental water samples using organic flocculation with skimmed milk, essentially as previously described by Hjelmsø et al. [61] and Calgua et al. [62]. In brief, skimmed milk (Difco, Franklin Lakes, NJ, USA) and Paragon Scientific™ Synthetic Sea Water (Thermo Fisher Scientific, Waltham, MA, USA) were added to a final concentration of 1% (*w*/*v*) and 3.2% (*w*/*w*), respectively. These mixtures were acidified to pH 3.5 with 1 M HCl, and then continuously mixed for 8 h at room temperature. The obtained flocculants sedimented overnight at room temperature, after which the supernatant was carefully removed with a vacuum pump (to avoid disturbing the sediment) and a residual volume of approximately 500 mL was centrifuged at 5500× *g*, for 45 min, at 4 °C. The supernatant was subsequently removed, and the obtained sediment was resuspended in 8 mL of 1/2 phosphate buffer (0.2 M Na_2_HPO_4_, 0.2 M NaH_2_PO_4_, pH 7.5).

All the samples analyzed were collected during October 2018, April 2019, July 2020, and October–November in 2020 in LMA (Figure 1). From each collection point, one sample was retrieved, corresponding to either solid particle-free wastewater (raw sewage influent) or environmental water samples, except for collection points 5 and 6, from which 3 and 2 samples were collected, respectively. Total DNA was extracted from 17 samples of VLP-enriched sediment using QIAamp^®^ DNA Mini Kit (Qiagen, Germantown, MD, USA) and eluted in 100 mL of AE buffer.

### 4.2. Primer Design and Touch-Down Multiplex-PCR Set-Up

The compilation of the different nucleotide (nt) and amino acids (aa) sequences used in this work was based on the selection of structural protein-coding (VP1-2) and whole-genomic data available in GenBank on 31 April 2020. These were either directly identified via their accession numbers or indirectly, singled-out because of similarity searches using BLASTn or using NCBI-Virus (https://www.ncbi.nlm.nih.gov/labs/virus/vssi/#/, accessed on 31 April 2020).

Primers were designed using a combined approach that included visual inspection of multiple alignments of nt sequences performed using the iterative G-INS-I method as implemented in MAFFT vs. 7 [63], and Primer Design-M [64]. A total of 24 primers were designed (Table 1) to allow the amplification of 11 HPyV sequences in two parallel sets of two nested touch-down multiplexed-amplification PCR protocols. In one case, PCR-A was set up to allow the amplification of HPyV1, 2, 5, 8, 9, 10, 11 using 4 pairs of primers in both the 1st and 2nd PCR rounds. On the other hand, PCR-B targeted the amplification of HPyV3, 4, 6, and 7 using 2 pairs of primers in both the 1st and 2nd PCR rounds. The thermal profiles of the two touchdown amplification protocols were the same for the 1st and 2nd PCR rounds. For the first round of PCR-A, the cycling conditions were 3 min at 95 °C, 10 cycles of 30 s at 95 °C, 30 s at 55 °C (with a decrease of 1 °C per cycle), 1 min and 15 s at 72 °C, 30 cycles of 30 s at 95 °C, 30 s at 45 °C, 1 min and 15 s at 72 °C and a final extension step of 7 min at 72 °C. The same thermal profile was used for the second round, except for the annealing temperature starting with 56 °C and stabilizing at 46 °C after the first 10 cycles. The same applies to PCR-B, with the cycling conditions used in the amplification protocol for both rounds being the same, with the annealing temperature for the first round starting at 63 °C/59 °C, reaching 53 °C/49 °C after the first 10 cycles, for the first and second round, respectively.

The different primers were designed so as not to include more than three degenerate positions, a minimal length of 19 nt, and a Tm not below 45 °C. All the primers were designed to allow the amplification, using nested-PCR protocols, of approximately overlapping and similar-sized DNA fragments (from 881 to 1175 nt) in the 2nd round of amplification.

### 4.3. DNA Cloning and Sequencing

PCR amplicons were either purified from agarose gels using the Zymoclean Gel DNA Recovery Kit (Zymo Research, Irvine, USA) and directly sequenced or cloned in a plasmid vector using either the CloneJET PCR Cloning Kit (Thermo Fisher Scientific, USA) or the NZY-A PCR Cloning Kit (NZYtech, Lisboa, Portugal), and NovaBlue (Novagen, Wisconsin, USA) competent cells, prepared essentially as described by Chung et al. [65]. Individual clones were selected, and their plasmid content was extracted (classical alkaline lysis miniprep) and analyzed. Recombinant plasmids containing inserts of exogenous DNA were sequenced using Sanger’s method (STABVIDA, Caparica, Portugal). Depending on the efficiency of clone recovery, a minimum of five and a maximum of 14 recombinant plasmid molecules were sequenced for each sample. In addition, a pooled sample containing 10 purified PCR fragments obtained using the PCR-A and PCR-B protocols from DNA extracted from six wastewater and one environmental sample, with a 5% spike of PhiX174 DNA, was analyzed by NGS. This was carried out using a DNA library of single indexing paired-end (150 bp × 2) fragments prepared using the NEBNext Ultra II Library Prep Kit (NEB, Ipswich, MA, USA), and analyzed on an MiSeq Illumina sequencer.

### 4.4. Nucleotide Sequence Analyses

Sanger sequence analysis was carried out using Bioedit 7.2 [66] while NGS low-quality filtering, adapter trimming, contig assembly, and taxonomic identification were carried out using the Genome Detective software [67].

Five nucleotide sequence datasets were analyzed during this study. One of these datasets included representative (*n* = 1 to 6) nt sequences of reference HPyV genomes as well as 65 sequences obtained in this study with a minimum length of 650 nt. Considering the preliminary taxonomic identification results for the sequences obtained during this study (using BLASTn/MegaBlast option), four HPyV-specific datasets (HPyV1, HPyV2, HPyV5, and HPyV6) were prepared. For those viruses for which a complete genomic sequence was available, protein datasets (including non-structural and structural protein) were also assembled.

Multiple alignments of nt sequences were performed using the iterative G-INS-I method as implemented in MAFFT vs. 7 [63], followed by their edition using GBlocks [68], allowing for less strict flanking positions. The multiple sequence alignments were systematically verified to ensure the correct alignment of homologous codons using BioEdit 7.2 [66].

The evolutionary information contained in each aligned dataset (phylogenetic signal) was assessed by likelihood mapping [69] using TREE-PUZZLE v5.3 [70]. Phylogenetic reconstructions were performed based on the maximum likelihood (ML) optimization criterion using the GTR + Γ + I as the best data-fitting substitution model, as suggested by IQ-TREE [71]. The stability of the obtained tree topologies was assessed both by bootstrapping with 1000 re-samplings of the original sequence data, and with an approximate likelihood ratio test (aLRT) with 1000 data iterations, carried out using IQ-TREE running on an Ubuntu server.

On the other hand, phylogeographic analyses carried out using a Bayesian statistical framework took advantage of the BEAST v1.10 software package [72]. All these phylogenetic reconstructions were carried out assuming a relaxed uncorrelated lognormal molecular clock model [73] as indicated by the ML Clock Test implemented in MEGA X, allowing for the accommodation of among-lineage rate variation, the GTR + Γ + I model, and a smooth skyline demographic prior [74]. Two independent Markov chain Monte-Carlo (MCMC) runs were performed using BEAST v1.10 until 1 × 10^8^ states were sampled, and 10% of which were discarded as burn-in. Chain convergence was verified using the Tracer software v1.7.1 (http://beast.bio.ed.ac.uk/tracer, accessed on 31 April 2020), which was also used to check for an adequate effective sample size higher than 200 after the removal of burn-in. The trees were logged on every 10,000th MCMC step, and the tree distribution was summarized using TreeAnnotator software v1.8.3 as a maximum clade credibility (MCC) tree, using median heights as the node heights in the tree. The FigTree v1.4.2 software was used to visualize the phylogenetic trees (http://tree.bio.ed.ac.uk/software/figtree/, accessed on 31 April 2020).

NeighborNet networks (NNn) were constructed using distance matrixes (corrected with the HKY model) using Splits Tree 4 software [75]. Principal coordinate analysis was performed using PCOORD (available at https://www.hiv.lanl.gov/content/sequence/PCOORD/PCOORD.html, accessed on 31 April 2020). Database sequence similarity searches were carried out with BLASTn (MegaBLAST option). The analyzing of putative genetic recombination events was carried out using RDP4 [76].

The nucleotide sequences obtained in the course of this work were deposited at the DDBJ database under accession numbers LC636333 to LC636405.

## Figures and Tables

**Table 1:** Primers used for detection of human polyomaviruses using touchdown nested-PCRs, expected amplicon sizes and number of primer mismatches for each HPyV type.

Reaction		Primers (5′-3′)	Number of Mismatches ^a^										
			HPyV1	HPyV2	HPyV3	HPyV4	HPyV5	HPyV6	HPyV7	HPyV8	HPyV9	HPyV10	HPyV11
PCR-A	1st round	HPyV1/2Fo: CTGCTCCTCAATGGATGTTGC (from 1489 to 1509) ^b^	0	0	-	-	10	-	-	7	9	7	17
		HPyV1/2Ro: ATCATRTCTGGGTCCCCTGGAAG (from 2606 to 2584) ^b^	0	0	-	-	8	-	-	4	7	7	8
		HPyV5Fo: GAAAATAGCTTGCTGCATTCTG (from 879 to 900) ^c^	35	15	-	-	0	-	-	7	10	10	14
		HPyV5Ro: GGGCCCACTCCATTCTCATC (from 1979 to 1960) ^c^	5	6	-	-	0	-	-	2	2	5	8
		HPyV8/9Fo: AGGAGGRGCAMATCAAAGAG (from 1198 to 1217) ^d^	3	2	-	-	9	-	-	0	0	3	10
		HPyV8/9Ro: ATAAAYTCTGACTTCTTCMAC (from 2336 to 2316) ^d^	6	5	-	-	4	-	-	0	0	10	8
		HPyV10/11Fo: CCTGGATAYAGACAMTTTSA (from 806 to 825) ^e^	9	17	-	-	10	-	-	11	13	0	0
		HPyV10/11Ro: TTAMAGGATAAGGATTTCTVA (from 2334 to 2313) ^e^	7	8	-	-	6	-	-	5	1	0	1
		Expected amplicon size (bp) ^f^	1113	1094	-	-	1101	-	-	1139	1136	1529	1474
PCR-A	2nd round	HPyV1/2Fi: GTACGGGACTGTAACACCTGC (from 1526 to 1546) ^b^	0	0	-	-	12	-	-	8	8	11	14
		HPyV1/2Ri: CCATACATAGGCTGCCCATC (from 2534 to 2515) ^b^	0	0	-	-	7	-	-	7	7	3	7
		HPyV5Fi: CAATCAAACCTAGTGAATCTG (from 951 to 971) ^c^	31	18	-	-	0	-	-	12	10	13	10
		HPyV5Ri: GGATCAGGACACCATACTTC (from 1859 to 1840) ^c^	7	5	-	-	0	-	-	7	5	6	4
		HPyV8/9Fi: GGWTTGTATGGTGATATAAC (from 1250 to1269) ^d^	7	6	-	-	9	-	-	0	0	5	7
		HPyV8/9Ri: ATTAAARTAYCTAGGTAGGCCTCT (from 2192 to 2170) ^d^	4	5	-	-	6	-	-	0	0	5	5
		HPyV10/11Fi: AGAGCTTTTTGGGARGCTKT (from 881 to 900) ^e^	8	7	-	-	14	-	-	6	10	0	1
		HPyV10/11Ri: CCCAGGCCTCYACWGGATAR (from 2055 to 2036) ^e^	3	6	-	-	5	-	-	4	5	0	1
		Expected amplicon size (bp) ^f^	989	985	-	-	909	-	-	943	940	1175	1136
PCR-B	1st round	HPyV3/4Fo: GGACGTGTTCAATAGAATTGC (from 980 to 1000) ^g^	-	-	0	0	-	10	8	-	-	-	-
		HPyV3/4Ro: CCAATGCCATTTTCATCCAA (from 2282 to 2263) ^g^	-	-	0	0	-	5	4	-	-	-	-
		HPyV6/7Fo: GACTCGGCCCAAGARTTGG (from 708 to 726) ^h^	-	-	9	13	-	0	0	-	-	-	-
		HPyV6/7Ro: GCACCTGTGGCTTCTGRGG (from 2220 to 2202) ^h^	-	-	7	6	-	0	0	-	-	-	-
		Expected amplicon size (bp) ^f^	-	-	1303	1318	-	1513	1513	-	-	-	-
PCR-B	2nd round	HPyV3/4Fi: CATCATATTACAATRCGGGG (from 1015 to 1034) ^g^	-	-	0	0	-	8	7	-	-	-	-
		HPyV3/4Ri: GTTTCCATTCTRTACAGCTC (from 1895 to 1876) ^g^	-	-	0	0	-	7	5	-	-	-	-
		HPyV6/7Fi: TGGCACTTCAAYTGTGGTTG (from 738 to 757) ^h^	-	-	27	18	-	0	0	-	-	-	-
		HPyV6/7Ri: WCCAATKACATCCAAGGGGC (from 1730 to 1711) ^h^	-	-	16	5	-	0	0	-	-	-	-
		Expected amplicon size (bp) ^f^	-	-	881	893	-	993	1002	-	-	-	-

K (G or T), M (A or C), R (A or G), S (C or G), W (A or T), Y (C or T), V (A, C or G). (HPyV1 = BK polyomavirus; HPyV2 = JC polyomavirus; HPyV3 = KI polyomavirus; HPyV4 = WU polyomavirus; HPyV5 = MC polyomavirus; HPyV8 = TS polyomavirus; HPyV11 = STL polyomavirus). ^a^ According to the GenBank reference sequences for each HPyV. The number of nucleotides in indels was counted as a mismatch. ^b^ Positions numbered according to the HPyV1 GenBank reference sequence NC_001538. ^c^ Positions numbered according to the HPyV5 GenBank reference sequence NC_010277. ^d^ Positions numbered according to the HPyV8 GenBank reference sequence NC_014361. ^e^ Positions numbered according to the HPyV10 GenBank reference sequence NC_018102. ^f^ Estimated according to the GenBank reference sequence for each HPyV. ^g^ Positions numbered according to the HPyV3 GenBank reference sequence NC_009238. ^h^ Positions numbered according to the HPyV6 GenBank reference sequence NC_014406.

**Table 2:** Distribution of the HPyV sequences analyzed in this work into different genetic types.

HPyV	Genotype/Subtype/Subgroup (% _total_; % _per Virus Species_)	Accession Number
PyV1	Ia (*n* = 2/2.74% _Total_; 13.33% _HPyV1_)	LC636348 ^a=5,c^, LC636350
	Ib1 (*n* = 11/15.07% _total_; 73.33% _HPyV1_)	LC636351, LC636353, LC636359, LC636362, LC636363, LC636364, LC636374, LC636378, LC636381, LC636385, LC636400
	III (*n* = 1/1.37% _total_; 6.67% _HPyV1_)	LC636340
	I-like (n = 1/1.37% _total_; 6.67% _HPyV1_)	LC636403
HPyV2	1A (*n* = 3/4.11% _total_; 18.75% _HPyV2_)	LC636370, LC636395, LC636401
	1B (*n* = 3/4.11% _total_; 18.75% _HPyV2_)	LC636349 ^a=5,c^, LC636360 ^a=13,c^, LC636404
	2A2 (*n* = 3/4.11% _total_; 18.75% _HPyV2_)	LC636358, LC636379, LC636380
	2B (*n* = 1/1.37% _total_; 6.25% _HPyV2_)	LC636396
	3A (*n* = 2/2.74% _total_; 12.50% _HPyV2_)	LC636377, LC636399 ^a=11,c^
	4 (*n* = 3/4.11% _total_; 18.75% _HPyV2_)	LC636357, LC636365 ^a=13,c^, LC636394
	9 (*n* = 1/1.37% _total_; 6.25% _HPyV2_)	LC636376
HPyV5	Africa (*n* = 8/10.96% _total_; 30.77% _HPyV5_)	LC636333, LC636335, LC636352, LC636355, LC636356, LC636384, LC636387 ^a=5,c^, LC636398
	Europe/North America (*n* = 17/23.29% _total_; 65.38% _HPyV5_)	LC636341, LC636342, LC636343, LC636347, LC636354, LC636369, LC636373, LC636375, LC636386, LC636388 ^a=5,c^, LC636389, LC636390, LC636391 ^a=5,c^, LC636392, LC636393, LC636397, LC636402
	Eu/NAm/Af (*n* = 1/1.37% _total_; 3.85% _HPyV5_)	LC636361
HPyV6	n.a. ^b^ (*n* = 16/21.91% _total_; 100.00% _HPyV6_)	LC636334, LC636336, LC636337, LC636338, LC636339, LC636344, LC636345, LC636346, LC636366, LC636367, LC636368, LC636371, LC636372, LC636382, LC636383, LC636405

^a^ The collection site of the sequences that were not phylogenetically analyzed is marked using the same annotation as the one used in the trees. ^b^ Not applicable. ^c^ Sequences typed using only BLASTn results.

## Data Availability

Data is contained within the article or Supplementary Material.

## Supplementary Materials

The following are available online at https://www.mdpi.com/article/10.3390/pathogens10101309/s1, Figure S1: Analysis of the phylogenetic relationships of 15 HPyV lineages (maximum likelihood) using the complete structural protein coding-sequence of references downloaded from the public genomic databases. Each sequence is identified by its accession number, HPyV type, country of origin, and, when possible, viral genotype and/or subtype. At the main tree branches ** indicates the support revealed by relevant aLRT and bootstrap values (≥75%). The bar indicates the average number of substitutions per site. Figure S2: Analysis of the sensitivity/specificity of amplification using the PCR-A and B touchdown protocols. MWM indicates the NZYtech molecular weight marker VI (NZYtech, Portugal). Lanes 1 and 6: 1st and 2nd round negative controls for PCR-A; lanes 2 and 7: analysis of the 1st (lane 2) and 2nd round (lane 7) amplification reactions of PCR-A using a positive control [a HPyV2 DNA extract (diluted 1:10,000) was used in the 1st-round (lane 2) and a 1:50 dilution of the 1st-round product as input in the 2nd-round (lane 7)]; lanes 3 and 8: 1st (lane 3) and 2nd (lane 8) round of a singleplex reaction using primers HPyV1/2Fo and HPyV1/2Ro or HPyV1/2Fi and HPyV1/2Fi, for the 1st and 2nd round, respectively. The input DNA was the same as the one mentioned for the reactions in lanes 2 and 7; lanes 4 and 9: negative control (1st and 2nd-round, respectively) for PCR-B; lanes 5 and 10: analysis of 1st (lane 5) and 2nd round (lane 10) amplification reactions of PCR-B using as input for the 1st round a diluted (1:10,000) HPyV2 DNA extract and as 1:50 dilution of the product obtain in the 1st round as input in the 2nd round (lane 10). The “*” indicates the expected band with approximately 1100 bp (1st round) and 990 (2nd round). Figure S3: Phylogenetic analysis of HPyV1 (a), HPyV2 (b), HPyV5 (c), and HPyV6 (d) sequences (maximum likelihood) considering a structural protein-coding genome region. At specific branches, the identity of the HPyV is indicated by their accession number and country of origin. For the analysis of HPyV1, HPyV2 and HPyV5, the different viral genotypes/subtypes, as defined previously [14,20], are indicated. The sequences obtained during this study are indicated in boldface. In the latter, the numbers indicated in superscript refer to the identity of the sample collection sites, as they are indicated in Figure S1. The consensus sequence generated by NGS is indicated by the arrow. At the main tree branches, the number of “*” indicates the support revealed by the different phylogenetic reconstruction methods used, assuming as relevant aLRT and bootstrap values ≥75%, as well as posterior probability values ≥0.80 when a Bayesian approach was further used to confirm the proposed phylogenetic clustering. The bars indicate the average number of substitutions per site. Figure S4: NeighborNet networks (A) and PCOORD (B) analyses of HPyV1 (A1/B1), HPyV2 (A2/B2), HPyV5 (A3/B3), and HPyV6 (A4/B4) sequences. The sequences obtained in the course of this study are indicated by their accession number. In the PCOORD graphs, the two first dimensions accounts for 50.30% (B1), 47.02% (B2), 73.95% (B3), and 40.20% (B4) if the total nucleotide differences, while the first 10 axes cover 90.60% (B1), 78.78% (B2), 90.88% (B3), and 67.75% (B4) of the dataset sequence variation. Table S1: Human polyomaviruses classification, isolation origin, and associated human diseases. Table S2: Analysis of the performance of the primers used for amplification of HPyV sequences in a singleplex vs. multiplex format.
