# Supplementary material for: Human Polyomaviruses (HPyV) in Wastewater and Environmental Samples from the Lisbon Metropolitan Area: Detection and Genetic Characterization of Viral Structural Protein-Coding Sequences
Source: Pathogens. 2021 Oct 12;10(10):1309. doi: 10.3390/pathogens10101309 (PMC8540013; doi:10.3390/pathogens10101309)
Supplement: Supplementary file 1 [file pathogens-10-01309-s001.zip › pathogens-1399809-supplementary.pdf]

Supplementary Materials

Supplementary Table S1 - Human polyomaviruses classification, isolation origin, and associated human diseases.

Virus	Genus	Species	Abbreviation	Genotypes/Subtypes/Subgroups[a]	Isolation sites	Associated clinical diseases
BKPyV[1]	*Betapolyomavirus*	*Human polyomavirus 1*	HPyV1	I (Ia, Ib1, Ib2, Ic), II, III, IV (IVa1, IVa2, IVb1, IVb2, IVc1, IVc2)	Urine, feces	Haemorrhagic cystitis, nephropathy
JCPyV[2]	*Betapolyomavirus*	*Human polyomavirus 2*	HPyV2	1 (1A, 1B), 2 (2A/C [2A1, 2A2], 2B, 2D [2D1, 2D2, 2D3], 2E), 3 (3A, 3B), 4, 6, 7 (7A, 7B [7B1, 7B2], 7C [7C1, 7C2]), 8 (8A, 8B), Eu-c	Brain tissue, urine	Progressive multifocal leukoencephalopathy
KIPyV[3]	*Betapolyomavirus*	*Human polyomavirus 3*	HPyV3	n.d.	Respiratory tract	None
WUPyV[4]	*Betapolyomavirus*	*Human polyomavirus 4*	HPyV4	I (Ia, Ib, Ic, Id), II, III (IIIa, IIIb, IIIc)	Respiratory tract	None
MCPyV[5]	*Alphapolyomavirus*	*Human polyomavirus 5*	HPyV5	Europe/North America, Africa, Asia, Oceania, South America	Skin cancer	Merkel cell carcinoma
HPyV6[6]	*Deltapolyomavirus*	*Human polyomavirus 6*	HPyV6	Worldwide, Asia	Healthy skin, skin tumors	Pruritic dermatitis
HPyV7[7]	*Deltapolyomavirus*	*Human polyomavirus 7*	HPyV7	n.d.	Healthy skin, skin tumors	Pruritic dermatitis
TSPyV[8]	*Alphapolyomavirus*	*Human polyomavirus 8*	HPyV8	n.d.	Skin lesions	Trichodysplasia spinulosa
HPyV9[9]	*Alphapolyomavirus*	*Human polyomavirus 9*	HPyV9	n.d.	Serum, urine	None
HPyV10[10]	*Deltapolyomavirus*	*Human polyomavirus 10*	HPyV10	n.d.	Diarrheic and non-diarrheic feces	None
STLPyV[11]	*Deltapolyomavirus*	*Human polyomavirus 11*	HPyV11	n.d.	Diarrheic and non-diarrheic feces	None
HPyV12[12,b]	n.d.	*Human polyomavirus 12*	HPyV12	n.d.	Gastrointestinal tract, liver	None
NJPyV[13]	*Alphapolyomavirus*	*Human polyomavirus 13*	HPyV13	n.d.	Muscle tissue	None
LIPyV[14]	*Alphapolyomavirus*	*Human polyomavirus 14*	HPyV14	n.d.	Blood, skin	None
QPyV[15,c]	n.d.	n.d.	HPyV15	n.d.	Feces	None

“n.d.” stands for “non determined”.

[1]BK polyomavirus; [2]John Cunningham polyomavirus; [3]Karolinska Institute polyomavirus.; [4]Washington University polyomavirus; [5]Merkel Cell Polyomavirus; [6]Human polyomavirus 6; [7]Human polyomavirus 7; [8]Trichodysplasia spinulosa polyomavirus; [9]Human polyomavirus 9; [10]Human polyomavirus 10; [11]Saint Louis polyomavirus; [12]Human polyomavirus 12; [13]New Jersey polyomavirus; [14]Lyon-IARC polyomavirus; [15]Quebec polyomavirus.

[a] Subtypes are indicated inside the parentheses while the respective subgroups are indicated inside square brackets.

[b] Excluded from the family on the ICTV 2019 report.

[c] Recently described, not yet featured on the current family *Polyomaviridae* taxonomy report (ICTV 2019).

Supplementary Table S2 - Analysis of the performance of the primers used for amplification of HPyV sequences in a singleplex vs multiplex format.

	1st-round					
	PCR-A				PCR-B	
	HPyV1/2	HPyV5	HPyV8/9	HPyV10/11	HPyV3/4	HPyV6/7
Site 2^{wastewater}	+	-	-	-	-	+
Site 7^{wastewater}	+	+	-	-	-	+
Site 10^{environmental}	-	-	-	-	-	-
Site 14^{environmental}	-	-	-	-	-	-
	2nd-round					
	PCR-A				PCR-B	
	HPyV1/2	HPyV5	HPyV8/9	HPyV10/11	HPyV3/4	HPyV6/7
Site 2^{wastewater}	+	-	-	-	-	+
Site 7^{wastewater}	+	+	-	-	-	+
Site 10^{environmental}	+	-	-	-	-	-
Site 14^{environmental}	-	-	-	-	-	-

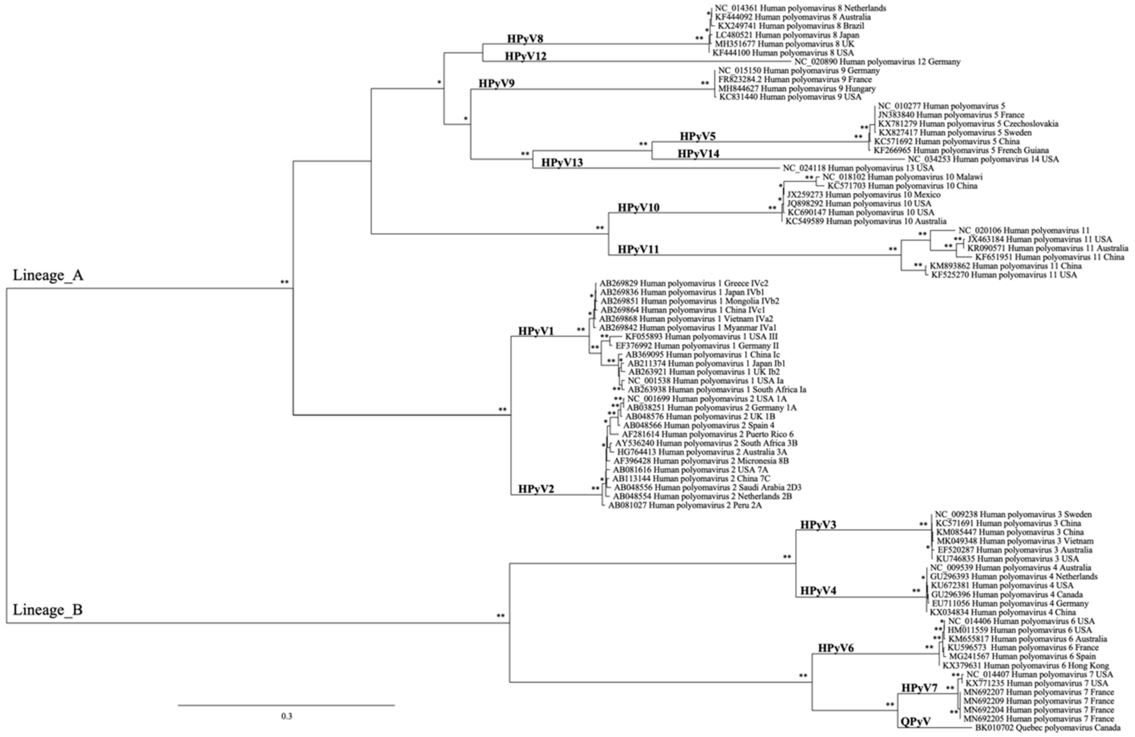

Supplementary Figure S1 - Analysis of the phylogenetic relationships of 15 HPyV lineages (maximum likelihood) using the complete structural protein coding-sequence of references downloaded from the public genomic databases. Each sequence is identified by its accession number, HPyV type, country of origin, and, when possible, viral genotype and/or subtype. At the main tree branches ** indicates the support revealed by relevant aLRT and bootstrap values (≥75%). The bar indicates the average number of substitutions per site.

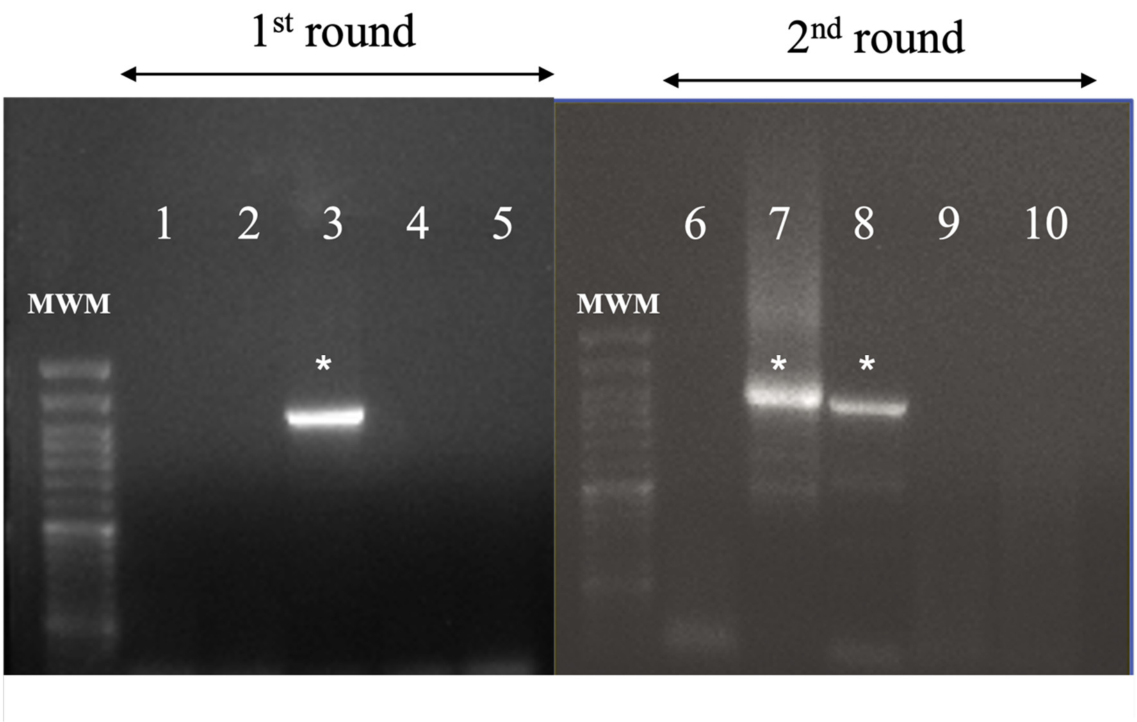

Supplementary Figure S2 - Analysis of the sensitivity/specificity of amplification using the PCR-A and B touchdown protocols. MWM indicates the NZYtech Molecular Weight marker VI (NZYtech, Portugal). Lanes 1 and 6: 1st and 2nd round negative controls for PCR-A; lanes 2 and 7: analysis of the 1st- (lane 2) and 2nd-round (lane 7) amplification reactions of PCR-A using a positive control [a HPyV2 DNA extract (diluted 1:10,000) was used in the 1st-round (lane 2) and a 1:50 dilution of the 1st-round product as input in the 2nd-round (lane 7)]; lanes 3 and 8: 1st- (lane 3) and 2nd- (lane 8) round of a singleplex reaction using primers HPyV1/2Fo and HPyV1/2Ro or HPyV1/2Fi and HPyV1/2Fi, for the 1st and 2nd round, respectively. The input DNA was the same as the one mentioned for the reactions in lanes 2 and 7; lanes 4 and 9: negative control (1st and 2nd-round, respectively) for PCR-B; lanes 5 and 10: analysis of 1st (lane 5) and 2nd-round (lane 10) amplification reactions of PCR-B using as input for the 1st-round a diluted (1:10,000) HPyV2 DNA extract and as 1:50 dilution of the product obtain in the 1st-round as input in the 2nd-round (lane 10). The "*" indicates the expected band with approximately 1100 bp (1st-round) and 990 (2nd-round).

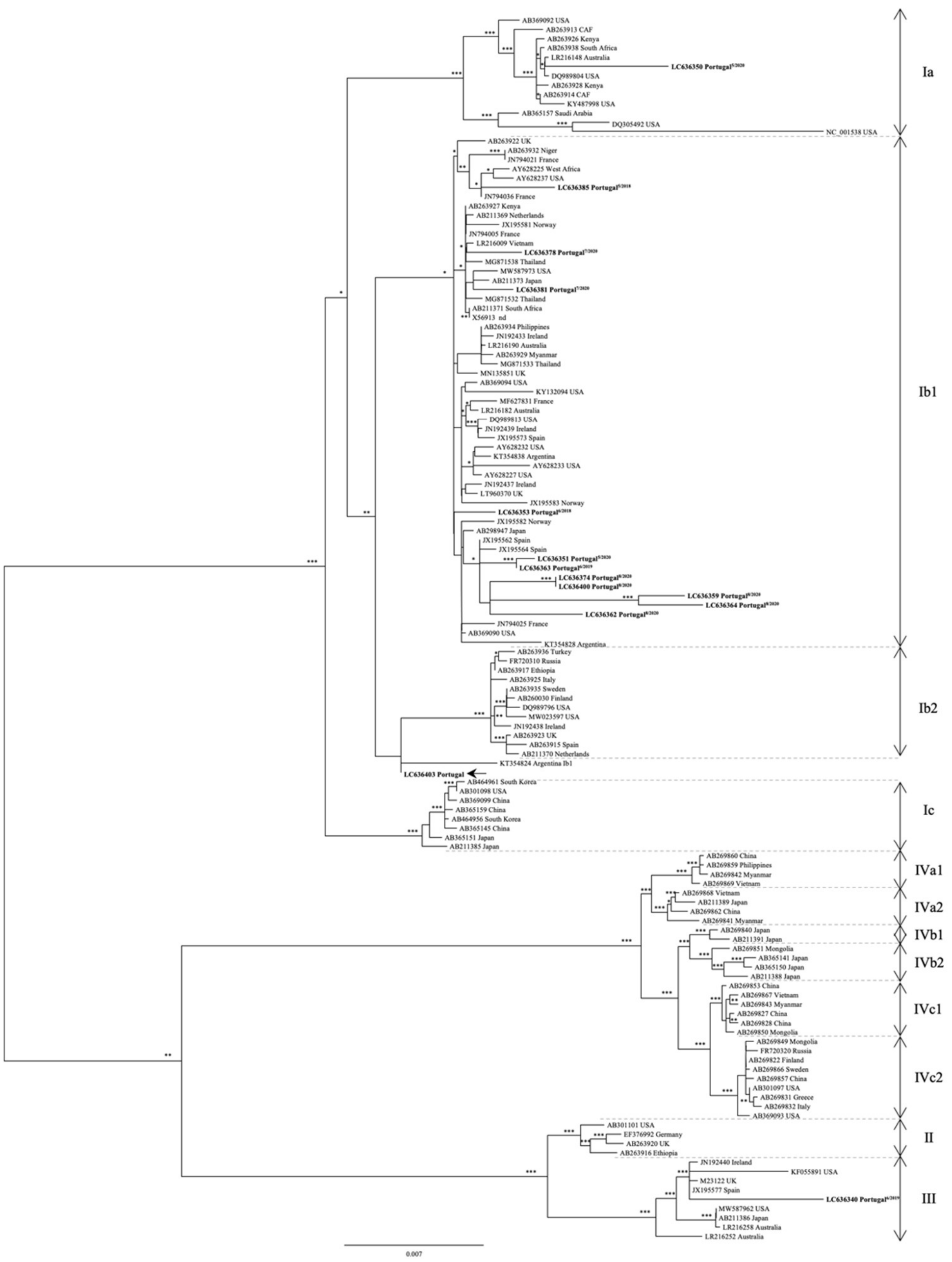

AB369092 USA
AB263913 CAF
AB263926 Kenya
AB263938 South Africa
LR216148 Australia
LC636350 Portugal
DQ989804 USA
AB263928 Kenya
AB263914 CAF
KY487998 USA
AB365157 Saudi Arabia
DQ305492 USA
NC_001538 USA
AB263922 UK
AB263932 Niger
JN794021 France
AY628225 West Africa
AY628237 USA
LC636385 Portugal
JN794036 France
AB263927 Kenya
AB211369 Netherlands
JX195581 Norway
JN794005 France
LR216009 Vietnam
LC636378 Portugal
MG871538 Thailand
MW587973 USA
AB211373 Japan
LC636381 Portugal
MG871532 Thailand
AB211371 South Africa
X56913 nd
AB263934 Philippines
JN192433 Ireland
LR216190 Australia
AB263929 Myanmar
MG871533 Thailand
MN135851 UK
AB369094 USA
KY132094 USA
MF627831 France
LR216182 Australia
DQ989813 USA
JN192439 Ireland
JX195573 Spain
AY628232 USA
KT354838 Argentina
AY628233 USA
AY628227 USA
JN192437 Ireland
LT960370 UK
JX195583 Norway
LC636353 Portugal
JX195582 Norway
AB298947 Japan
JX195562 Spain
JX195564 Spain
LC636351 Portugal
LC636363 Portugal
LC636374 Portugal
LC636400 Portugal
LC636359 Portugal
LC636364 Portugal
LC636362 Portugal
JN794025 France
AB369090 USA
KT354828 Argentina
AB263936 Turkey
FR720310 Russia
AB263917 Ethiopia
AB263925 Italy
AB263935 Sweden
AB260030 Finland
DQ989796 USA
MW023597 USA
JN192438 Ireland
AB263923 UK
AB263915 Spain
AB211370 Netherlands
KT354824 Argentina Ib1
LC636403 Portugal
AB464961 South Korea
AB301098 USA
AB369099 China
AB365159 China
AB464956 South Korea
AB365145 China
AB365151 Japan
AB211385 Japan
AB269860 China
AB269859 Philippines
AB269842 Myanmar
AB269869 Vietnam
AB269868 Vietnam
AB211389 Japan
AB269862 China
AB269841 Myanmar
AB269840 Japan
AB211391 Japan
AB269851 Mongolia
AB365141 Japan
AB365150 Japan
AB211388 Japan
AB269853 China
AB269867 Vietnam
AB269843 Myanmar
AB269827 China
AB269828 China
AB269850 Mongolia
AB269849 Mongolia
FR720320 Russia
AB269822 Finland
AB269866 Sweden
AB269857 China
AB301097 USA
AB269831 Greece
AB269832 Italy
AB369093 USA
AB301101 USA
EF376992 Germany
AB263920 UK
AB263916 Ethiopia
JN192440 Ireland
KF055891 USA
M23122 UK
JX195577 Spain
LC636340 Portugal
MW587962 USA
AB211386 Japan
LR216258 Australia
LR216252 Australia
Ia
Ib1
Ib2
Ic
IVa1
IVa2
IVb1
IVb2
IVc1
IVc2
II
III
0.007

Sup_Fig_3a

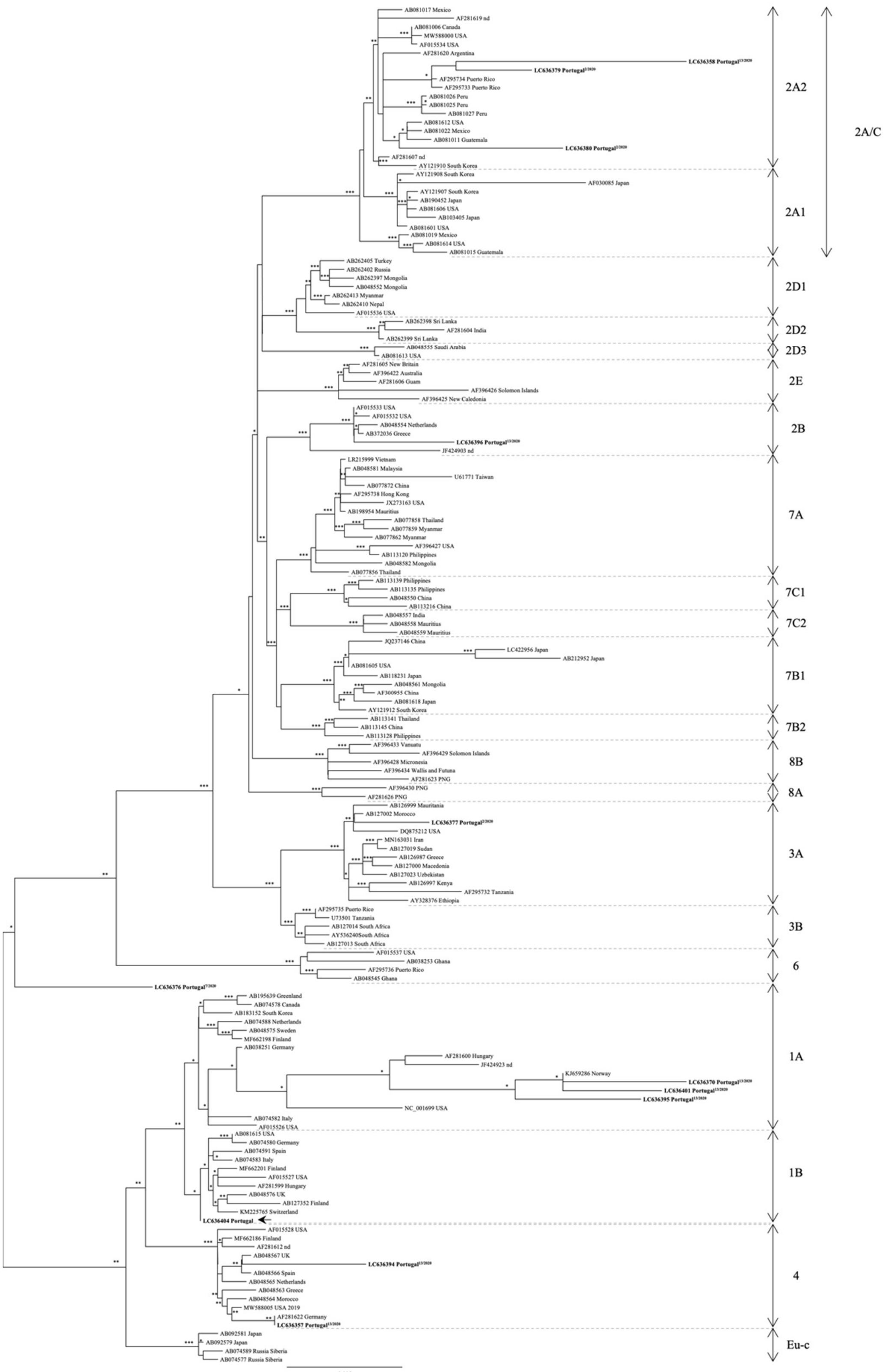
2A2
2A/C
2A1
2D1
2D2
2D3
2E
2B
7A
7C1
7C2
7B1
7B2
8B
8A
3A
3B
6
1A
1B
4
Eu-c
0.005

Sup_Fig_3b

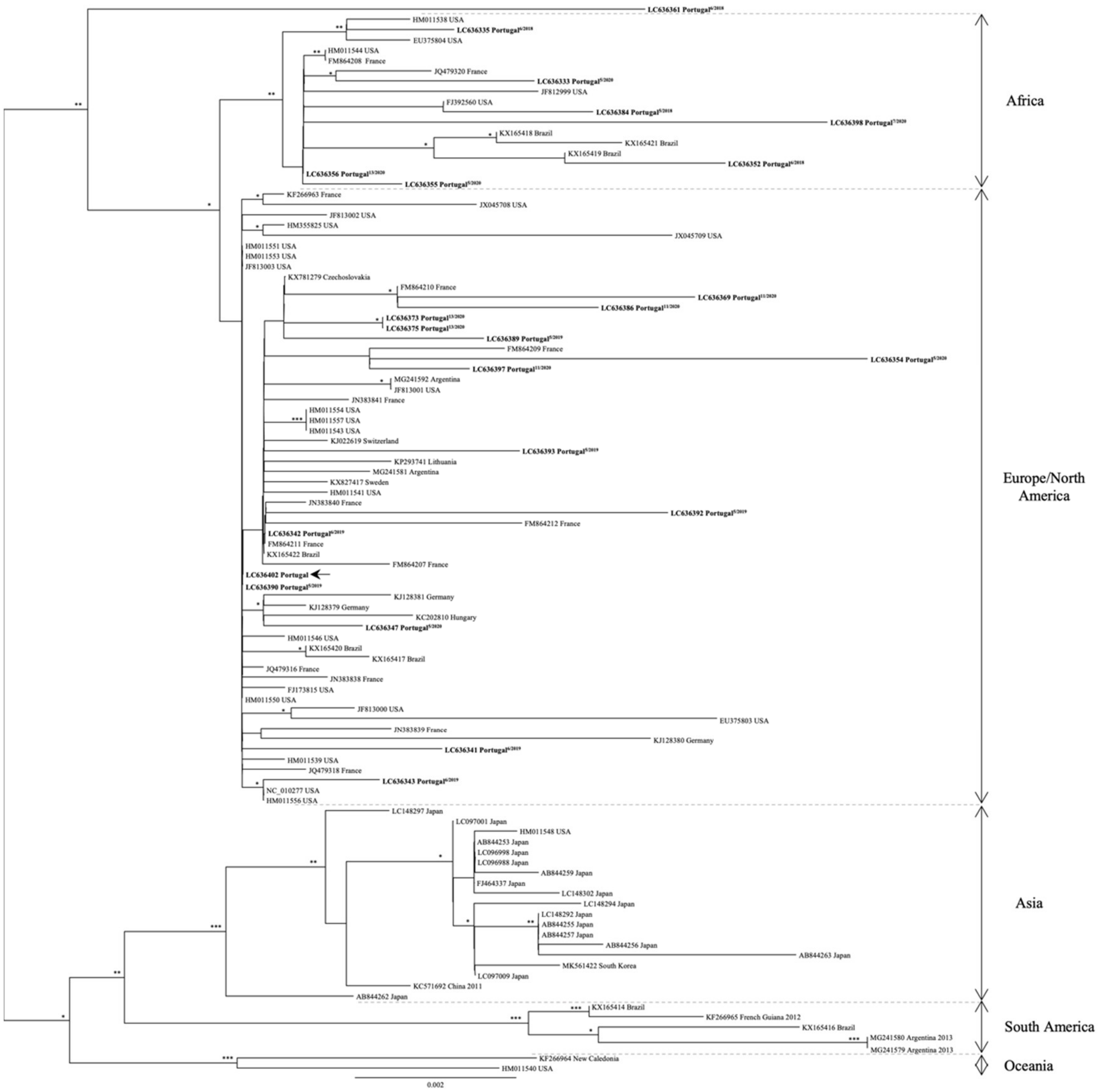
LC636361 Portugal6/2018
HM011538 USA
LC636335 Portugal6/2018
EU375804 USA
HM011544 USA
FM864208 France
JQ479320 France
LC636333 Portugal5/2020
JF812999 USA
FJ392560 USA
LC636384 Portugal5/2018
LC636398 Portugal7/2020
KX165418 Brazil
KX165421 Brazil
KX165419 Brazil
LC636352 Portugal6/2018
LC636356 Portugal11/2020
LC636355 Portugal5/2020
KF266963 France
JX045708 USA
JF813002 USA
HM355825 USA
JX045709 USA
HM011551 USA
HM011553 USA
JF813003 USA
KX781279 Czechoslovakia
FM864210 France
LC636369 Portugal11/2020
LC636386 Portugal11/2020
LC636373 Portugal12/2020
LC636375 Portugal12/2020
LC636389 Portugal5/2019
FM864209 France
LC636354 Portugal5/2020
LC636397 Portugal11/2020
MG241592 Argentina
JF813001 USA
JN383841 France
HM011554 USA
HM011557 USA
HM011543 USA
KJ022619 Switzerland
LC636393 Portugal5/2019
KP293741 Lithuania
MG241581 Argentina
KX827417 Sweden
HM011541 USA
JN383840 France
LC636392 Portugal5/2019
FM864212 France
LC636342 Portugal6/2019
FM864211 France
KX165422 Brazil
FM864207 France
LC636402 Portugal
LC636390 Portugal5/2019
KJ128381 Germany
KJ128379 Germany
KC202810 Hungary
LC636347 Portugal5/2020
HM011546 USA
KX165420 Brazil
KX165417 Brazil
JQ479316 France
JN383838 France
FJ173815 USA
HM011550 USA
JF813000 USA
EU375803 USA
JN383839 France
KJ128380 Germany
LC636341 Portugal6/2019
HM011539 USA
JQ479318 France
LC636343 Portugal6/2019
NC_010277 USA
HM011556 USA
LC148297 Japan
LC097001 Japan
HM011548 USA
AB844253 Japan
LC096998 Japan
LC096988 Japan
AB844259 Japan
FJ464337 Japan
LC148302 Japan
LC148294 Japan
LC148292 Japan
AB844255 Japan
AB844257 Japan
AB844256 Japan
AB844263 Japan
MK561422 South Korea
LC097009 Japan
KC571692 China 2011
AB844262 Japan
KX165414 Brazil
KF266965 French Guiana 2012
KX165416 Brazil
MG241580 Argentina 2013
MG241579 Argentina 2013
KF266964 New Caledonia
HM011540 USA
0.002
Africa
Europe/North America
Asia
South America
Oceania

Sup_Fig_3c

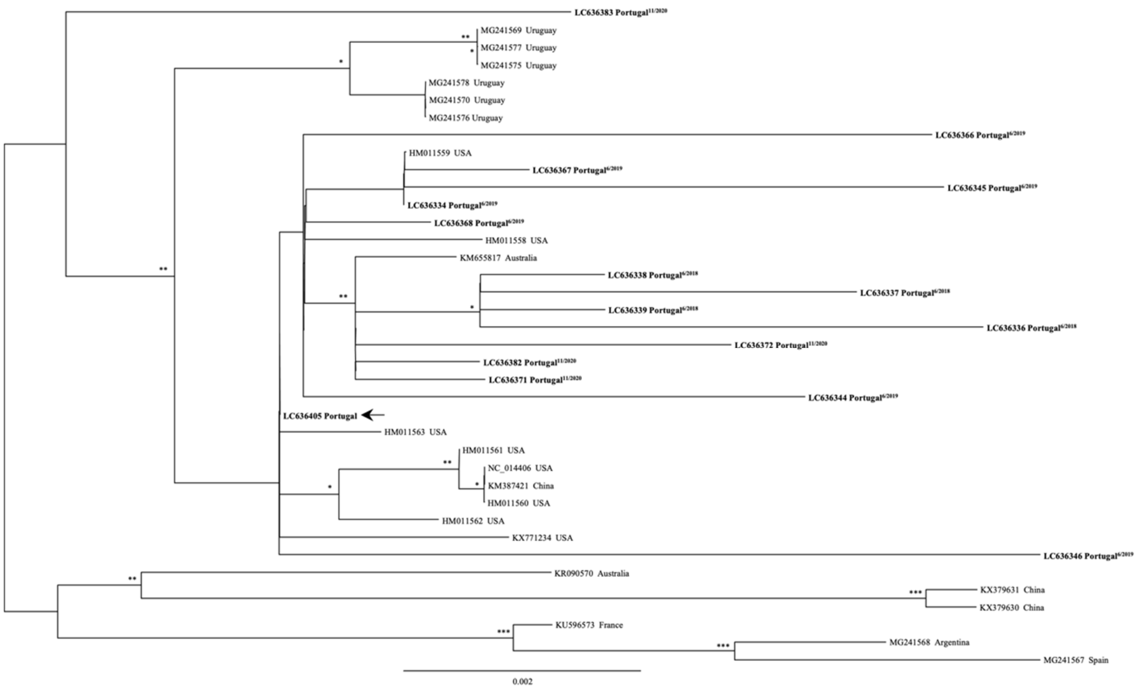

Sup_Fig_3d

Supplementary Figure S3 - Phylogenetic analysis of HPyV1 (a), HPyV2 (b), HPyV5 (c), and HPyV6 (d) sequences (maximum likelihood) considering a structural protein-coding genome region. At specific branches, the identity of the HPyV is indicated by their accession number and country of origin. For the analysis of HPyV1, HPyV2 and HPyV5, the different viral genotypes/subtypes, as defined previously [14,20], are indicated. The sequences obtained during this study are indicated in boldface. In the latter, the numbers indicated in superscript refer to the identity of the sample collection sites, as they are indicated in Fig.1. The consensus sequence generated by NGS is indicated by the arrow. At the main tree branches, the number of "*" indicates the support revealed by the different phylogenetic reconstruction methods used, assuming as relevant aLRT and bootstrap values ≥75%, as well as posterior probability values ≥0.80 when a Bayesian approach was further used to confirm the proposed phylogenetic clustering. The bars indicate the average number of substitutions per site.

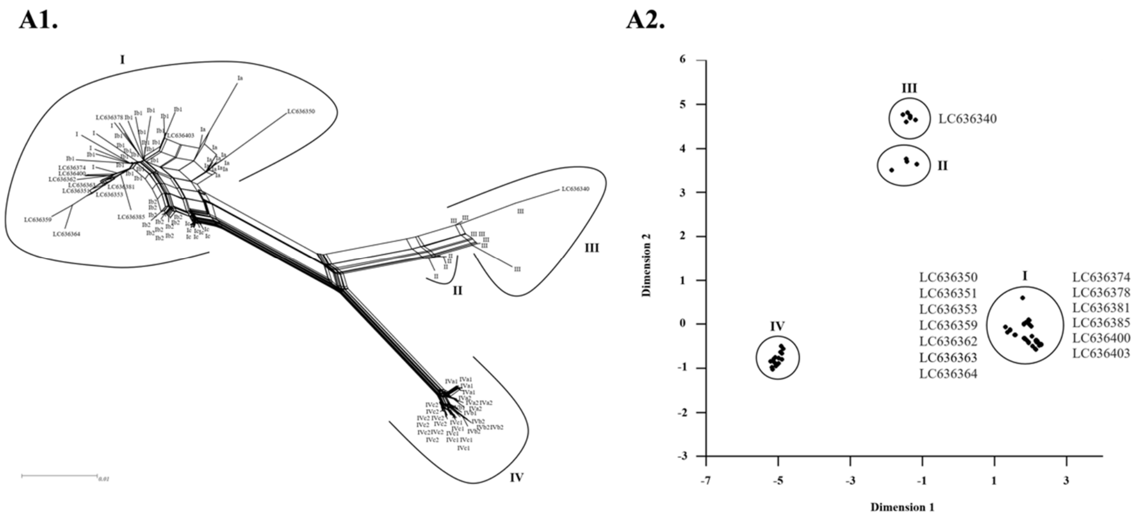

Sup_Fig_4a

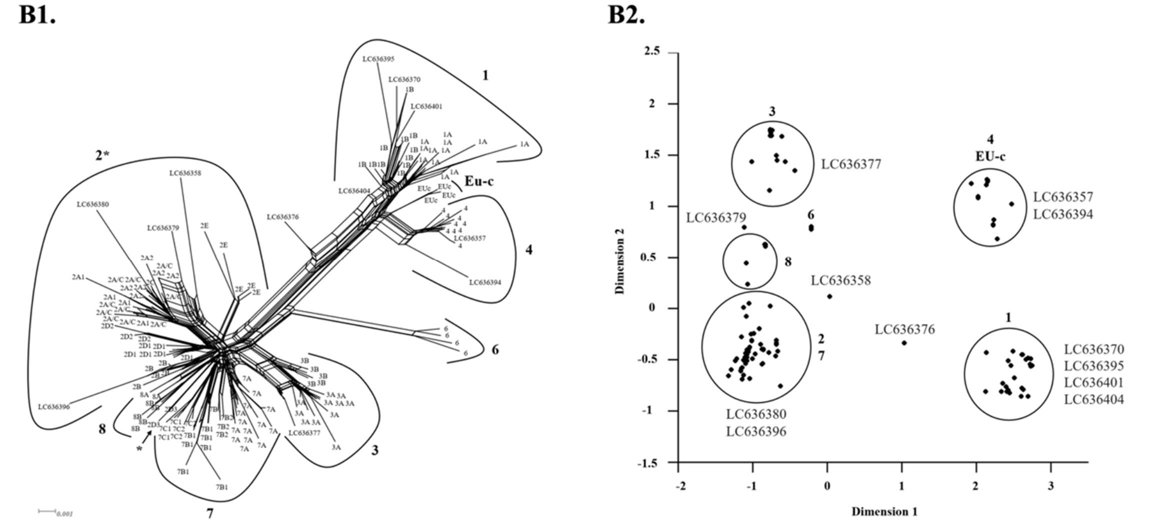

Sup_Fig_4b

C1.

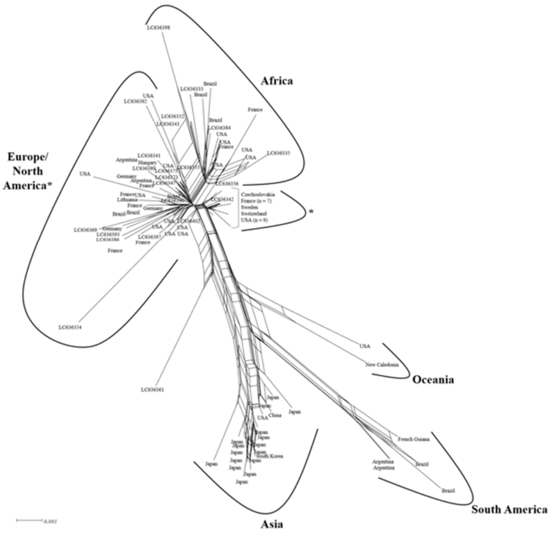

C2.

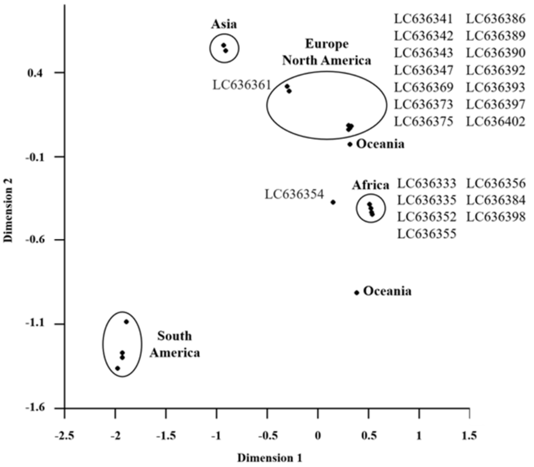

Sup_Fig_4c

D1.

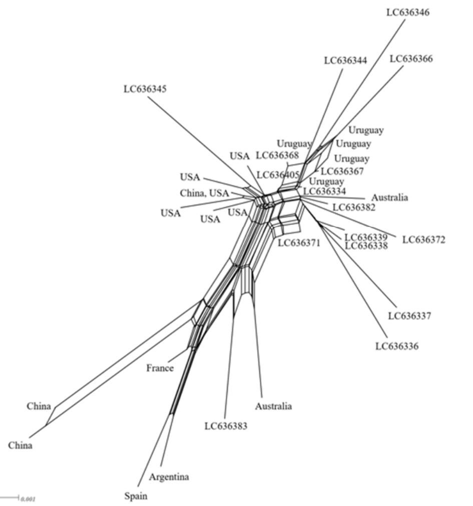

D2.

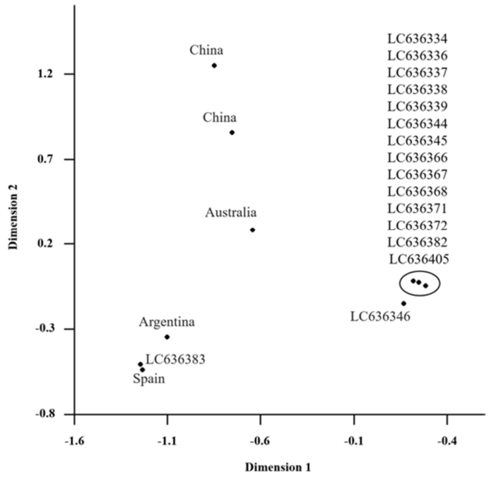

Sup_Fig_4d

Supplementary Figure S4 - NeighborNet networks (A) and PCOORD (B) analyses of HPyV1 (A1/B1), HPyV2 (A2/B2), HPyV5 (A3/B3), and HPyV6 (A4/B4) sequences. The sequences obtained in the course of this study are indicated by their accession number. In the PCOORD graphs, the two first dimensions accounts for 50.30% (B1), 47.02% (B2), 73.95% (B3), and 40.20% (B4) if the total nucleotide differences, while the first 10 axes cover 90.60% (B1), 78.78% (B2), 90.88% (B3), and 67.75% (B4) of the dataset sequence variation.
